# Prognostic factors for persistent symptoms in adults with mild traumatic brain injury: an overview of systematic reviews

**DOI:** 10.1186/s13643-023-02284-4

**Published:** 2023-07-20

**Authors:** Julien Déry, Béatrice Ouellet, Élaine de Guise, Ève-Line Bussières, Marie-Eve Lamontagne

**Affiliations:** 1 School of Rehabilitation Sciences, Université Laval, Pavillon Ferdinand-Vandry, local 2475, 1050, avenue de la Médecine, Québec, QC G1V 0A6 Canada; 2 Centre interdisciplinaire de recherche en réadaptation et intégration sociale (Cirris), 525, boul. Wilfrid-Hamel, Québec, QC G1M 2S8 Canada; 3 Department of Psychology, Université de Montréal, Montréal, Canada; 4 Research Institute of the McGill University Health Centre (RI-MUHC), Montréal, Canada; 5 Centre de recherche interdisciplinaire en réadaptation du Montréal métropolitain (CRIR), Montréal, Canada; 6 Department of Psychology, Université du Québec à Trois-Rivières, 3007 Michel-Sarrazin, 3600 rue Sainte-Marguerite, Trois-Rivières, QC G9A 5H7 Canada

**Keywords:** Overview, Systematic review, Prognosis, Concussion, Post-concussion symptoms

## Abstract

**Background:**

Mild traumatic brain injury (mTBI) is an increasing public health problem, because of its persistent symptoms and several functional consequences. Understanding the prognosis of a condition is an important component of clinical decision-making and can help to guide the prevention of persistent symptoms following mTBI. The prognosis of mTBI has stimulated several empirical primary research papers and many systematic reviews leading to the identification of a wide range of factors. We aim to synthesize these factors to get a better understanding of their breadth and scope.

**Methods:**

We conducted an overview of systematic reviews. We searched in databases systematic reviews synthesizing evidence about the prognosis of persistent symptoms after mTBI in the adult population. Two reviewers independently screened all references and selected eligible reviews based on eligibility criteria. They extracted relevant information using an extraction grid. They also rated independently the risk of bias using the ROBIS tool. We synthesized evidence into a comprehensive conceptual map to facilitate the understanding of prognostic factors that have an impact on persistent post-concussion symptoms.

**Results:**

From the 3857 references retrieved in a database search, we included 25 systematic reviews integrating the results of 312 primary articles published between 1957 and 2019. We examined 35 prognostic factors from the systematics reviews. No single prognostic factor demonstrated convincing and conclusive results. However, age, sex, and multiple concussions showed an affirmatory association with persistent post-concussion outcomes in systematic reviews.

**Conclusion:**

We highlighted the need for a comprehensive picture of prognostic factors related to persistent post-concussion symptoms. We believe that these prognostic factors would guide clinical decisions and research related to prevention and intervention regarding persistent post-concussion symptoms.

**Systematic review registration:**

PROSPERO CRD42020176676

**Supplementary Information:**

The online version contains supplementary material available at 10.1186/s13643-023-02284-4.

## Background

Incidence of mild traumatic brain injury (mTBI) has increased in the last decades [1–3], and persistent symptoms associated with this condition have received increasing attention [4]. In fact, even if most of the symptoms of an mTBI resolve in a few days or weeks after the trauma [5], 5 to 20% of individuals encounter persistent physical, cognitive, and behavioral persistent symptoms [6]. Such symptoms, like headaches, difficulty concentrating, fatigue, and dizziness [7] that persist after mTBI, can have important impacts on day-to-day activities and lead to functional consequences (such as workplace fatigue and inability to maintain workload/standards) [8, 9]. To prevent these consequences, it is important to acutely recognize the factors that can influence the persistence of symptoms [6]. The definition of persistent symptoms after mTBI caused controversy over the years [5, 10]. However, a recent expert consensus was reached in 2021 by Lagacé-Legendre et al. [11] regarding the definition of mTBI persistent symptoms as a whole, which includes the actual symptoms, their frequency, and duration: “presence of any symptom that cannot be attributed to a preexisting condition (e.g., preexisting mental health problems) and that appeared within hours of an mTBI, that is still present every day 3 months after the trauma, and that has an impact on at least one sphere of a person’s life.” Persistent symptoms after mTBI are both complex and non-specific, because many can overlap with other conditions (e.g., low back pain and whiplash-associated disorders), which highlights the need to identify the subgroups of the mTBI population that might benefit from specific and timely interventions [12]. Acutely identifying these factors would help healthcare providers to better appraise the prognosis of individuals with mTBI and identify the patients that have many factors associated with a poor prognostic to intervene before the symptoms become chronic [13, 14].

Indeed, understanding a condition’s prognostic factors, defined as “a variable associated with a subsequent outcome such as disability among people with a disease or health condition” [14], is recognized as an important component of clinical decision-making [13, 15]. Patients with many factors associated with a poor prognosis should arguably be considered for more in-depth evaluation and targeted intervention in acute and subacute stages of the condition to prevent symptom persistence. Persistent symptoms could thus be reduced by identifying and addressing earlier prognostic factors, such as comorbidities (e.g., depression, anxiety) [12, 16]. As such exhaustive listing of prognostic factors has not been produced yet, it is difficult for clinicians and researchers to take in consideration the range of potential factors in clinical decision-making for assessing the prognosis of the patient and prioritizing them.

Multiple systematic reviews synthesized information about this population and prognostic factors associated with various outcomes. The World Health Organization (WHO) Collaborating Centre Task Force has produced twelve systematic reviews related to the prognosis for mTBI between 2004 and 2016 [2, 4, 5, 12, 17–23]. These reviews mainly synthesized studies employing longitudinal design to identify time to recovery and prognostic factors affecting recovery or symptom persistence [19]. The WHO reviews identified various predictors of prolonged symptoms or associated with slower recovery, such as financial compensation, being married, being off work due to the injury, post-injury symptoms of nausea or memory problems, and many more [19]. In addition, some authors have systematically reviewed the evidence about particular prognostic factors including sex [18], age [24, 25], and biomarkers [26, 27], and others were interested in specific outcomes related to mTBI, such as cognitive and psychiatric outcomes [5, 22], adding to the complex portrait of potential predictors of persistent symptoms. Despite extensive evidence, the breadth and scope of prognostic factors associated with persistent post-concussion symptoms have not yet been determined nor integrated into a comprehensive model such as the post-concussional syndrome model proposed by Hou et al. [28] which organized the predictors in larger categories of prognostic factors. Our objective is to synthesize evidence from systematic reviews on the nature of factors that affect the risk of persistent symptoms in adults with mTBI.

## Methods

We conducted an overview of systematic reviews (OvSR) following the principles of the Cochrane Handbook’ [29] and other recent methodological papers [30–32]. An OvSR is a rigorous approach to mapping evidence of a large body of literature in a given area [33, 34]. The aim of OvSR is not to report individual systematic review summaries, but it should aim to synthesize across included systematic review evidence to bring new insights to existing evidence [33]. It is also used to provide an accessible summary of evidence to support decision-making by clinicians, policymakers, and developers of clinical guidelines [35]. Despite their increasing popularity in healthcare research over the past years, there are currently no systematically developed reporting guidelines for OvSR [35, 36]. However, we conducted this OvSR in consultation with the PRISMA 2020 statement [37] (Supplemental file 1). This review has been registered in the PROSPERO database (CRD42020176676). Details about the specific methods used have been published previously [38].

### Criteria for selecting reviews for inclusion

We included reviews that employed a systematic review or meta-analysis design explicitly describing a systematic search, i.e., a search strategy and article selection process precisely presented (e.g., with a PRISMA flow diagram), with enough detail to be reproducible. We included reviews that targeted multiple populations (e.g., children and adults). However, we only analyzed the primary studies that were conducted with adults since the overview only targets this population. We did not restrict our inclusion process based on a specific setting, context, or specific eligible time period. We included reviews that highlight at least one prognostic factor related to the course of persistent post-concussion symptoms, defined for this OvRS as Légacé-Legendre et al. [11] stated. We also included reviews that did not specifically target post-concussion symptoms, but only the results related to outcomes after 3 months were integrated into this synthesis. We excluded narrative, non-systematic reviews, editorials/commentaries, conference abstracts, grey literature, and thesis. We also excluded reviews about moderate, severe, or non-traumatic brain injuries.

### Search methods for identification of reviews

Our search strategy included the two main concepts: “adults with mTBI/concussion” and “systematic reviews.” An example of a search strategy in MEDLINE (Ovid) is displayed in Supplemental file 2. We searched in Cochrane Library (Wiley), MEDLINE (Ovid), CINAHL (EBSCO), Embase (Elsevier), PsycINFO (Ovid), and Epistemonikos for systematic reviews published in peer-reviewed journals without date restrictions. We validated our strategy by consulting search filters such as those reported by the InterTASC Information Specialists’ Sub-Group [39].

### Selecting systematic reviews for inclusion

Once duplicates were removed, two reviewers independently screened all the titles and abstracts of all references imported from the databases in the Covidence online software [40]. They read the full text to include papers that were potentially relevant based on the eligibility criteria. The reviewers discussed disagreements until consensus, and a third member of the team was consulted if necessary.

### Managing overlapping systematic reviews

We took into consideration that systematic reviews may include the same primary studies. Thus, we created a citation matrix to visually demonstrate the number of overlapping reviews and we calculated the “corrected covered area” (CCA) [41]. We calculated the CCA as a measure of overlap by dividing the frequency of repeated occurrences of the primary studies in other reviews by the product of primary studies and reviews, and this product is reduced by the number of primary studies. A CCA value lower than 5 can be considered as a slight overlap, whereas values greater than or equal to 15 can be considered as a very high overlap [41].

### Data collection

We first extracted basic information from all selected reviews in a predefined grid, such as the title, authors, year of publication, designs of the primary studies, population studied, number of primary studies included, and purpose of the reviews. We then extracted from the reviews information related to prognostic factors, such as participant inclusion/exclusion criteria, sample size, and prognostic factors associated with outcomes related to persistent symptoms [13]. Our extraction focused on the outcomes related to persistent post-concussion symptoms, according to the definition proposed by Lagacé-Legendre et al. [11]. We limited our extraction to information presented in the included systematic reviews, so we did not examine the primary research studies [29]. Two independent reviewers (JD, BO) extracted data, and information was compared to reach a consensus.

### Summarizing data

We organized the information from the reviews into a comprehensive model about prognostic factors. We used the model proposed by Hou et al. [28] which organized the predictors in predisposing, precipitating, and perpetuating factors. Narrative descriptions and tabular forms complement the data and provide a comprehensive understanding of the prognostic factors. Systematic reviews of prognostic factors present their results in a form which does not permit re-analysis of primary data, so we did not quantify the prognostic effects in a risk prediction model [13, 42]. Given the goal of our study, we chose to gather all outcomes related to persistent post-concussion symptoms and not to run a secondary analysis on the specific outcomes.

### Quality of included reviews

Two assessors rated the risk of bias in the included reviews using the Risk of Bias in Systematic Reviews (ROBIS) tool [43]. ROBIS is useful and reliable for systematic reviewers to identify areas where bias may be introduced into systematic review methods: study eligibility criteria, identification and selection of studies, data collection and study appraisal, and synthesis and findings [43]. Both assessors (JD and BO) scored the risk of bias of the reviews as low, high, or unclear concerns, and these scores are displayed in a table and a figure as suggested by Whiting et al. [43]. We did not exclude reviews based on their risk of bias evaluations, but we took in consideration the scores attributed for each review in the synthesis.

## Results

**Fig. 1:**
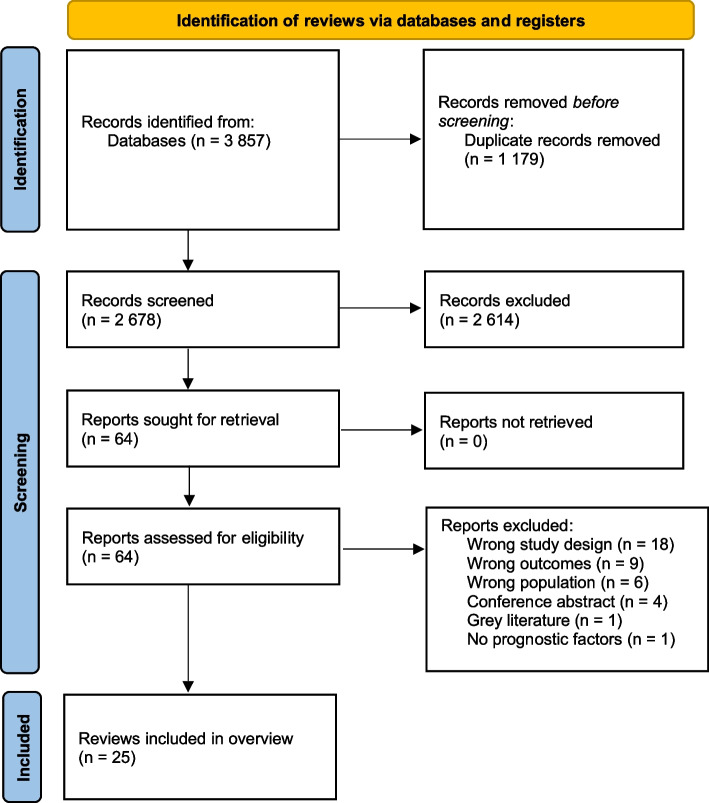
PRISMA flow diagram [37]

We retrieved 3857 records from the database search. Once duplicates were removed, we screened 2678 references, and we assessed 64 full-text articles for eligibility. We finally included 25 systematic reviews in our OvSR [2, 5, 17–19, 22, 24–27, 44–58]. The details of the selection process are presented in the PRISMA flow diagram (Fig. 1). We counted a total of 312 primary studies relevant to our objective, which were published between 1957 and 2018. This number excludes studies on pediatric populations included in some reviews [18, 19, 22, 50, 54, 56]. We present the characteristics of the reviews included in Table 1.

**Table 1:** Characteristics of the systematic reviews included

**Authors, year of publication**	**Nb of primary articles retained for the OvSR**	**Design of the studies included**	**Population**	**Purpose of the review**	**ROBIS assessment**
**Begaz et al., 2006** [44]	7/11	Prospective cohort studies	Adults with mTBI	Assess the predictive ability and potential clinical utility of known serum biochemical markers on the outcome of PCS in patients with closed head injury and mild TBI	HR
**Belanger et al., 2005** [45]	39/39	Not specified	Adults and adolescents with mTBI	Determine the magnitude of impairment in mTBI participants across multiple cognitive domains	HR
**Belanger et al., 2010** [46]	8/8	Not specified	Adult and adolescent athletes with mTBI	Determine the magnitude of impairment in those participants with multiple concussions across multiple cognitive domains	HR
**Biagianti et al., 2020** [47]	9/9	Longitudinal cohort and case-control studies	Adults with mTBI	Integrate findings from longitudinal studies that investigated across the spectrum of neuroimaging modalities the changes within the first twelve months following a mTBI, with the goal of identifying possible predictors or biomarkers of persistent PCS	HR
**Boyle et al., 2014** [17]	3/3	Retrospective cohort studies	Military with mTBI	Synthesize the best available evidence on the course and prognosis of mTBI in the military population	LR
**Cancelliere et al., 2014** [48]	4/4	Cohort studies	Workers with mTBI	Synthesize the best available evidence on RTW after mTBI	LR
**Cancelliere et al., 2016** [18]	12/16	Control trials, cohort, and case-control studies	Adults and children with mTBI	Determine sex differences in the recovery and prognosis after mTBI in adults and children	HR
**Carroll et al., 2004** [19]	53/120^a^	Cohort studies, randomized control trials, systematic reviews, cross-sectional studies, and case series or variant designs	Adults and children with mTBI	Produce a best-evidence synthesis on the epidemiology (incidence, risk, and prevention), diagnosis, treatment, and prognosis of mTBI	LR
**Carroll et al., 2014** [5]	20/21	Randomized control trials, cohort, and case-control studies	Adults with mTBI	Synthesize the best available evidence on objective outcomes after adult mTBI	LR
**Cassidy et al., 2014** [2]	20/23	Cohort and non-randomized experimental studies	Adults with mTBI (excluding sport/military injuries)	Update the WHO findings on course and prognosis in adults with respect to self-reported outcomes	LR
**Finkbeiner et al., 2016** [49]	15/23	Retrospective studies and qualitative studies	Adult athletes with concussion	Summarize the current literature addressing chronic (> 3 months) psychiatric changes, including emotional and behavioral domains (depression, anxiety, substance abuse, and challenging behaviors) with sport concussion in adults	HR
**Godbolt et al., 2014** [22]	2/8	Cohort studies and randomized control trials	Adults and children with mTBI	Synthesize the best available evidence regarding the risk of dementia and chronic cognitive impairment after mTBI	LR
**Khong et al., (2016)** [50]	9/10	Case-control	Adults and children with mTBI	Review the evidence for the use of DTI parameters in the human brain as a diagnostic tool for and predictor of PCS after a mTBI	HR
**King, 2014a** [24]	20/20	Retrospective studies, prevalence studies of risk factors, and outcome with/without matched controls	Adults with mTBI (16–65 years old)	Review the studies which have examined working age mTBI patients at 18+ months post-injury and examined the relationship between age or gender and permanent PCS	HR
**King, 2014b** [25]	16/16	Prevalence, prospective follow-up, explorative follow-up, and epidemiological studies	Adults with mTBI (16–65 years old)	Systematically review the literature to identify and examine those studies which have investigated working-age patients with prolonged PCS (i.e., at 12–18 months post-injury)	HR
**Ludwig et al., 2020** [51]	2/4	Prospective longitudinal study and longitudinal cohort study	> 16 years old individuals with mTBI	Examine the association between sleep during the acute stage (< 2 weeks) of a concussion and long-term post-concussive outcomes	LR
**Manley et al., 2017** [52]	47/47	Retrospective, case series, case-control, cross-sectional, cross-sectional surveys, cohort studies, and surveys	Adults with sport-related concussion	Address the current state of the scientific evidence about the prevalence, risk factors, and causation of possible long-term sequelae like chronic traumatic encephalopathy and other neurodegenerative diseases, with respect to sports concussion	LR
**Mercier et al., 2018a** [27]	21/29	Retrospective, prospective cohort studies, and randomized control trials	Adults with mTBI	Determine the prognostic value of S-100b protein to identify patients with post-concussion symptoms after a mTBI	LR
**Mercier et al., 2018b** [26]	7/10	Cohort studies	Adults with mTBI	Determine the prognostic value of neuron-specific enolase to predict post-concussion symptoms following mTBI	LR
**Merritt et al., 2019** [53]	10/37	Cross-sectional, epidemiology, repeated measures, retrospective and case series studies, systematic review, and meta-analysis	Adults with mTBI	Examine sex differences in concussion, or mTBI outcome, updating previous critical reviews of the literature	HR
**Ofoghi et al., 2020** [54]	14/19	Retrospective or prospective, observational, case-cohort, cohort, non-randomized studies	Individuals with mTBI	Evaluated the current literature examining the structural and functional neuroimaging correlates and pain processing network differences that could contribute to the persistence of pain following mTBI	HR
**Puig et al., 2020** [55]	10/14	Prospective or retrospective cohort studies	Individuals > 16 years old with mTBI	Evaluate the current literature examining the use of MRI-based brain-connectivity mapping techniques including DTI and/or rs-fMRI to predict clinical outcomes following acute mTBI	LR
**Silverberg et al., 2015** [56]	18/26	Prospective inception cohort studies	Adults and children with mTBI	Identify and evaluate multivariable prognostic models for mTBI and determine which pre-, peri-, and early post-injury variables have independent prognostic value in the context of multivariable models	LR
**Sullivan et al., 2016** [57]	4/5	Cohort and cross-sectional studies	Adults with mTBI	Determine how the term resilience has been used in adult mTBI research	HR
**Zhu et al., 2019** [58]	6/9	Cohort and case-control studies	Adults with mTBI	Explore the association between DTI findings and cognitive function following mTBI using a meta-analysis	HR

*DTI* diffusion tensor imaging, *HR* high risk, *LR* low risk, *MRI* magnetic resonance imaging, *PCS* postconcussion symptoms, *rs-fMRI* resting-state functional magnetic resonance imaging, *RTW* return to work, *WHO* World Health Organization

^a^This review does not present the results of all articles included

### Overlapping systematic reviews

The CCA was calculated as follows: (365 − 312)/((312 × 25) − 312) = 0.007. Of the 312 primary publications, only 1 was included in 4 reviews, 7 were included in 3 reviews, and 36 were included in 2 reviews. The citation matrix is available in Supplemental file 3. Considering the very slight overlap found, we did not conduct further analysis to avoid double-counting of primary results.

### Risk of bias

**Fig. 2:**
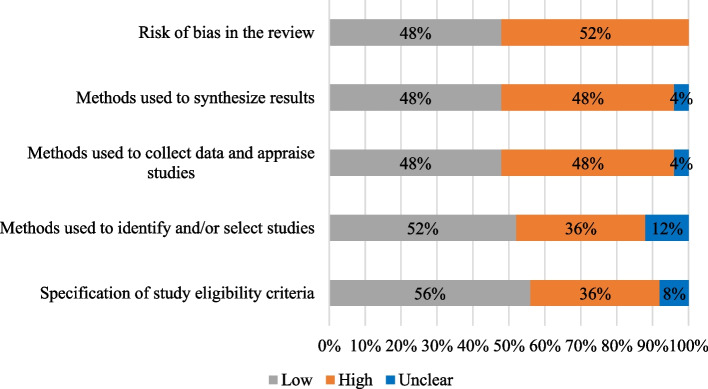
Graphical presentation of ROBIS results from all studies

We evaluated the risk of bias with the ROBIS tool of the 25 systematic reviews included. We displayed the summarized results of this assessment in Fig. 2. Of the 25 reviews, 12 were considered as low risk of bias, and 13 were assessed as high risk of bias. Details of ROBIS results in a tabular form are available in Supplemental file 4.

### Prognostic factors

Overall, 35 individual prognostic factors were extracted from the systematic reviews. These factors are presented in Table 2 and definitions retrieved from the systematic reviews or from another relevant source of information when available in Table 3. We summarized the results by documenting their association with the outcomes of interest predicted (persistent symptoms and their related consequences). We divided the factors into three types of association with outcomes:

- (+) Reviews that described a positive association between the factor and at least one outcome of mTBI
- (+/−) Reviews that described limited or mixed evidence of an association between the factor and at least one outcome of mTBI
- (−) Reviews that concluded no association between the factor and at least one outcome of mTBI

**Table 2:** Association between prognostic factors and outcomes described in the reviews

**Prognostic factors**	**(+)**	**(+/−)**	**(−)**	**Total number of reviews**
Biomarkers	**3** (50, 54, 58)	**9** (2, 5, 26, 27, 44, 47, 52, 55, 56)		**12**
Sex	**3** (24, 25, 53)	**4** (5, 18, 19, 56)	**1** (48)	**8**
Age	**3** (2, 24, 25)	**3** (19, 48, 56)		**6**
Multiple concussions	**2** (46, 49)	**3** (19, 52, 56)	**1** (22)	**6**
Negative perceptions/expectations	**3** (2, 5, 22)	**1** (56)		**4**
Somatic complaints	**2** (2, 48)	**2** (19, 56)		**4**
Litigation/financial compensation	**2** (2, 19)	**1** (45)		**3**
Mental health (pre-injury)	**2** (2, 56)	**1** (19)		**3**
Extracranial injuries	**2** (17, 48)	**1** (56)		**3**
Level of education	**2** (2, 48)	**1** (56)		**3**
Anxiety/depression	**2** (2, 56)		**1** (5)	**3**
PTSD	**2** (17, 19)		**1** (5)	**3**
LOC		**1** (5)	**2** (2, 56)	**3**
Nausea/vomiting	**1** (48)	**1** (19)		**2**
Marital status	**1** (2)	**1** (19)		**2**
Memory of the injury event	**1** (2)	**1** (5)		**2**
Physical health (pre-injury)	**1** (56)	**1** (19)		**2**
PTA		**2** (2, 56)		**2**
Mechanism of injury		**1** (19)	**1** (56)	**2**
Acute stress disorder (ASD)	**1** (19)			**1**
Baseline mental/physical health	**1** (2)			**1**
Degree of trait of resilience	**1** (57)			**1**
Baseline noise sensitivity	**1** (2)			**1**
Job independence/decision-making latitude at work	**1** (48)			**1**
Gross national product of the country	**1** (2)			**1**
Pre-injury fatigue	**1** (2)			**1**
Being a student		**1** (19)		**1**
Being sober/intoxicated in ED		**1** (56)		**1**
Quality of sleep		**1** (51)		**1**
Prior neurological problems		**1** (19)		**1**
Life stressors		**1** (19)		**1**
Complicated mTBI		**1** (19)		**1**
Sensory motor test		**1** (56)		**1**
Cognitive/neuropsychological tests		**1** (56)		**1**
Glasgow Coma Scale score			**1** (56)	**1**

*ED* Emergency department, *LOC* Loss of consciousness, *mTBI* Mild traumatic brain injury, *PTA* Post-traumatic amnesia, *PTSD* Post-traumatic stress disorder

**Table 3:** Definitions of the prognostic factors

**Prognostic factors**	**Definitions**
Biomarkers	Almost any measurement reflecting an interaction between a biological system and a potential hazard, which may be chemical, physical, or biological [59]: Serum protein level (S100b, S100A1b, NSE), genetic determinants (apolipoprotein E e4 allele), and brain structural changes (DTI, MRS, PET, task-based FMR, resting-state fMRI, CT, MRI).
Sex	Refers to a set of biological attributes in humans and animals, usually categorized as female or male [60].
Multiple concussions	Number of concussions, frequency of concussions, or history of concussion [22, 49, 52].
Negative perceptions/expectations	Expectations of the potential negative impact of head injuries or negative head injury perceptions [2, 5].
Somatic complaints	Physical symptoms experience early post-injury, such as head pain, hearing problems, arm numbness, headache, low back pain, and mid-back pain [2, 48].
Litigation/financial compensation	Individuals seeking financial compensation, involved in litigation process, or lawyer involvement [2, 45].
Mental health (pre-injury)	History of psychological, mental, or psychiatric health problems before the trauma event [2].
Extracranial injuries	Concurrent injury during the trauma (measured by Abbreviated Injury Severity Scale) [17, 48].
Anxiety/depression	Baseline or early post-injury anxiety/depression measured with BAI/BDI or HADS [2, 5].
PTSD	Diagnosis of PTSD (measured by Clinician-Administered PTSD Scale and the Post-traumatic Stress Symptom Scale) [5].
LOC	Presence of LOC following the trauma (maximum duration included differs from < 15 or < 30 min) [2].
Nausea/vomiting	Nausea and vomiting on hospital admission [48].
Marital status	Single, engaged, separated, divorced, widowed, etc. [2].
Physical health (pre-injury)	Pre-existing physical limitations [19].
PTA	Presence of PTA or its duration (maximum < 24 h) [2].
Mechanism of injury	Referring to the cause of injury and the resulting physiological or structural damage: direct impact, sudden or rapid acceleration and deceleration (e.g., motor vehicle injury), penetrating injury, and blast injury [61].
ASD	DSM-V diagnosis criteria: exposure to actual or threatened death, serious injury, sexual violation, and presence of nine or more symptoms in any of the five categories of intrusion, negative mood, dissociation, avoidance, and arousal [62].
Baseline mental/physical health	Measured with SF-36, a short-form health survey yields an eight-scale profile of scores as well as physical and mental health summary measures [63].
Degree of trait of resilience	The term “trait of resilience” is defined by the two core concepts of personal adaptation and adversity and is an adaptive process, which may fluctuate and thus be modifiable [57].
Baseline noise sensitivity	Potential physical post-concussion symptoms [2].
Job independence/decision-making latitude at work	Student, homemaker, professional/semiprofessional, and management categories were defined as occupations offering more independence and opportunity for decision-making, when compared with the clerical, sales and service, manual labor, and skilled crafts and trades occupations [48].
Gross national product (GNP) of the country	Countries were classified using the World Bank Atlas as either high-income (GNP > $10,066) or middle-/low-income countries (GNP < $10,065) [2].
Pre-injury fatigue	Not described in the reviews.
Being sober/intoxicated in ED	Being sober (versus intoxicated with alcohol) in the emergency department (ED) following mTBI [56].
Quality of sleep	Sleep disturbance assessed using subjective or objective measures < 2 weeks following concussion (baseline) (e.g., Insomnia Severity Index, Pittsburgh Sleep Quality Index) [51].
Prior neurological problems	Not described in the reviews
Life stressors	Not described in the reviews
Complicated mTBI	Mild complicated TBI defined as GCS 13–15 with focal brain lesion, depressed skull fracture, or both [19].
Sensory-motor test	Not described in the reviews
Cognitive/neuropsychological tests	Not described in the reviews

*DTI* Diffusion tensor imaging, *MRS* Magnetic resonance spectroscopy, *PET* Positron emission tomography, *fMR(I)* Functional magnetic resonance (imaging), *CT* Computed tomography, *BAI/BDI* Beck Anxiety/Depression Inventory, *HADS* Hospital Anxiety and Depression Scale, *PTSD* Post-traumatic stress disorder, *LOC* Loss of consciousness, *PTA* post-traumatic amnesia, *ASD* Acute stress disorder, *DSM* Diagnostic and Statistical Manual of Mental Disorders, *GCS* Glasgow Coma Scale

**Fig. 3:**
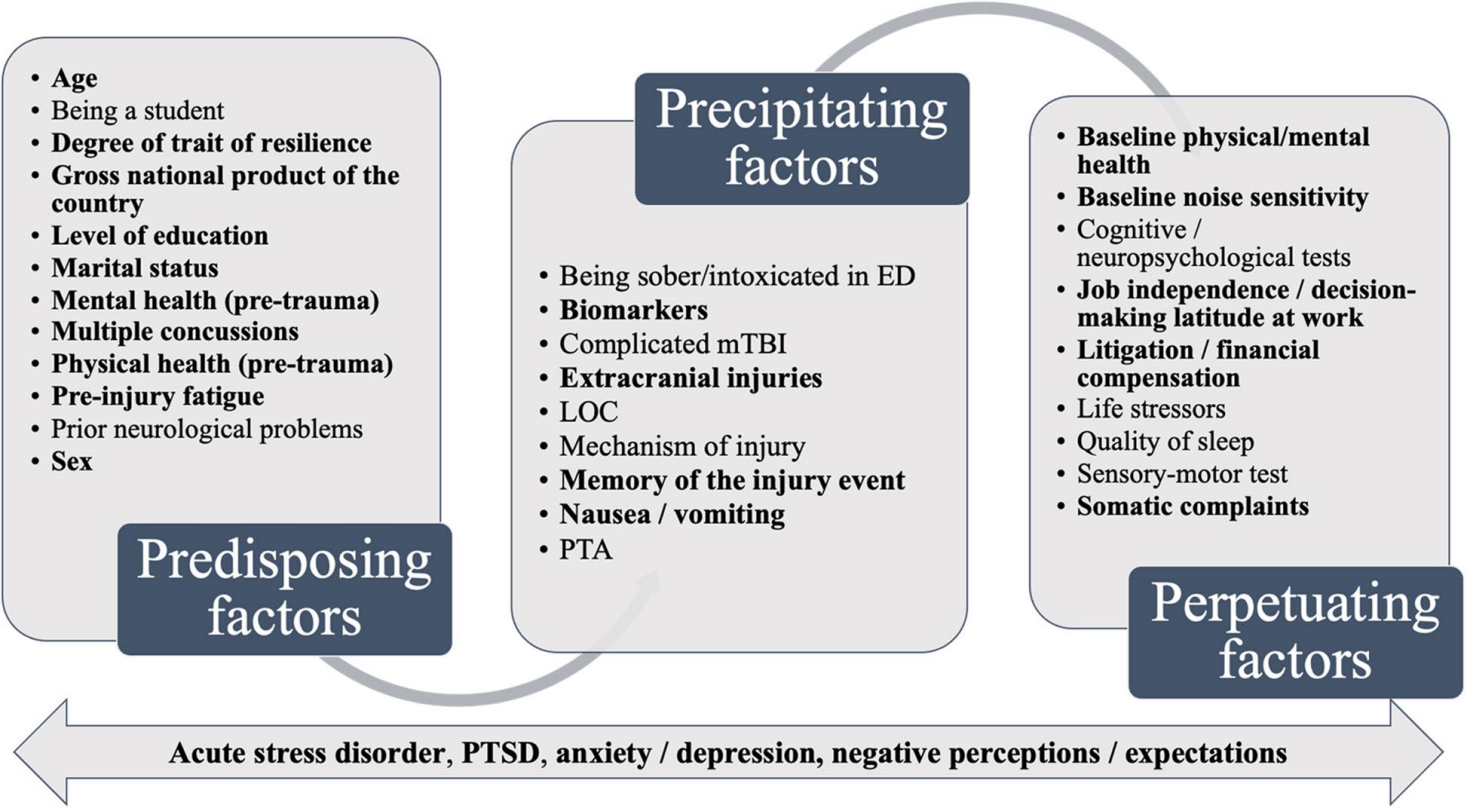
Model of prognostic factors associated with persistent post-concussion symptoms

We mapped the prognostic factors into the model developed by Hou et al. [28] also used by Rickards et al. [64] (Fig. 3). We highlighted in bold type the 23 factors that were described being associated with persistent post-concussion outcomes in at least one systematic review. We added factors that could not be included in only one category, so we integrated them overlapping the model.

The *predisposing factors* are characterized by premorbid attributes that may predispose people with mTBI to experiencing persistent PCS. Our overview highlighted that many personal factors, such as age, sex, level of education, and marital status, were found to be prognostic factors for persistent PCS. Personality traits of resilience have shown to be a “protective” factor including in “predisposing” factor because resilience tends to be related to fewer long-term PCS in adults. Premorbid mental and physical problems have been reported to be relevant factors resulting in poor outcomes, whereas having prior neurological problems found limited evidence as a prognostic factor. Sustaining multiple concussions has been shown in systematic reviews to be one of the most frequently mentioned factors associated with the presence of PCS after 3 months and more.

The *precipitating* factors are considered as characteristics of the injury or related to the event. The most studied factors have been related to the presence of specific biomarkers, such as brain structural changes, protein serum level, or the presence of a specific gene allele. With 9 reviews presenting mixed results, we found no clear consensus in systematic reviews about their association with poorer outcomes 3 months after mTBI in the adult population. Having suffered an extracranial injury, having memory of the event, or experiencing nausea or vomiting seem to be prognostic factors based on some reviews included. Other precipitating factors related to the mTBI, such as the mechanism of the injury, the presence of LOC or PTA after the trauma, and being intoxicated tend to show more limited results in the literature.

The *perpetuating factors* represent all the other characteristics that contribute to the maintenance of the PCS. Being involved in litigation or receiving financial compensation has been reported to be associated to the persistence of PCS, and it could delay return to work after mTBI. The presence of pain (e.g., back or neck pain), headaches, or other somatic complaints experienced by adults with mTBI have been demonstrated to predict worse outcomes. One systematic review highlighted that individuals having job independence and decision-making latitude at work can be a “protective” factor that helps RTW.

Psychiatric disorders (i.e., ASD, PTSD, anxiety, and depression) and negative perceptions of the injury or symptoms can influence the onset or contribute to the maintenance of the persistent PCS.

### Outcomes predicted

We focused our overview on the outcomes measured 3 months or more after the mTBI. Outcomes considered had to be related to at least one post-concussion symptom or its functional consequences [11]. One review highlighted only outcomes related to return to work (RTW) [48], but all the other reviews showed results of mixed post-concussion symptoms and outcomes (including cognitive or neuropsychological functioning outcomes). Some other outcomes were also considered as they are related to “impact on patient’s quality of life and functioning” [11], such as measures of quality of life [2, 27, 56, 57], social integration [51], and substance abuse [49]. Given the goal of our study and the small number of reviews focusing on those outcomes, we chose not to run a secondary analysis on those specific effects.

## Discussion

There are increasing interests in the population and in the research about mTBI prevention, diagnosis, and prognosis to improve treatment and clinical decision-making [5, 19, 20, 65]. Multiple systematic reviews synthesized information about this population and prognostic factors associated with various outcomes. We aimed to present a synthesis of systematic reviews concerning any variable associated with persistent post-concussion symptoms after 3 months. We provided a comprehensive overview of the state of the evidence available concerning the 35 prognostic factors that have been systematically reviewed. Premorbid characteristics, such as age, sex, prior concussions, and mental/physical problems, need to have special attention having stronger evidence that demonstrated a relation with poor outcome 3 months post-injury. Having somatic complaints (e.g., headaches, neck or back pain) after the injury and negative perceptions or expectations related to the recovery were also associated with persistent symptoms following mTBI. However, we could not conclude a clear prognostic relation regarding physiological characteristics after mTBI such as the presence of specific biomarkers.

Other authors had interests in reviewing, understanding, and identifying prognostic or risk factors associated with persistent problems related to a specific condition, such as whiplash injury [66–68], neck pain [69, 70], orthopedic trauma [71], and all TBI [72].

Walton et al. [66] found 9 significant predictors for persistent problems following whiplash injury, such as no postsecondary education, female gender, history of previous neck pain, baseline neck pain intensity greater than 55/100, presence of neck pain at baseline, presence of headache at baseline, catastrophizing, whiplash-associated disorder grade 2 or 3, and no seat belt in use at the time of collision. Scholten-Peeters et al. [67] showed that of over 100 different prognostic factors examined, only one (high initial pain intensity) demonstrated a strong evidence association with persisting symptoms after whiplash-associated disorders. Finally, a meta-review of Sarrami et al. [68] presented five associated factors with the prognosis for people with whiplash injury, which were post-injury pain, disability and anxiety, catastrophizing, compensation and legal factors, and early use of healthcare. Walton et al. [69] suggested that the prognosis of neck pain of various causes is generally poor, and there are relatively few factors that allow high or moderate confidence in their use as predictors of outcome. Clay et al. [71] reported strong evidence supporting the association of female sex, older age, high pain intensity, preinjury anxiety or depression, and fewer years of education with persistent pain outcomes following acute orthopedic trauma. Willemse-van et al. [72] reported in their review that older age, pre-injury unemployment, pre-injury substance abuse, and more severe disability at rehabilitation discharge were strong predictors for long-term disability after TBI. Hence, these conditions seem to have similar prognosis factors for persistent symptoms. Older age, female gender, and presence of somatic complaints after the event, such as the intensity of pain, can be considered as general prognostic factors to examine from the outset.

Most prognostic factors highlighted in these reviews and in our overview are premorbid characteristics or early-on symptoms, which are variables related to the injury that cannot be modified. It is however possible that prioritizing individuals with a higher number of prognosis factors would lead to better outcomes in care. We also discuss that interventions could target some perpetuating factors, by reducing life stressors, improving the quality of sleep and focusing on realist expectations, and helping to manage symptomatic complaints. Although there is no clear evidence on the effectiveness of interventions for reducing persistent post-concussion symptoms [73–76], educational and behavioral interventions seem promising like a multidimensional psychoeducative and counseling intervention (SAAM) based on a biopsychosocial model, addresses misconception and perception of mTBI recovery and postconcussion symptoms through up-to-date psychoeducation, and provides reassurance and counseling about recovery and motivation for change [77]. Thus, post-acute recommended interventions tend to match with these objectives, such as education regarding normalizing symptoms, expected outcomes and positive recovery, technique to manage stress, and gradual return to activities and life roles [78]. While commonly used treatment remains educational and reassurance early after the injury, these interventions showed mixed results concerning effectiveness [76, 79]. The current evidence tend to suggest psychological and rehabilitative strategies [76], such as cognitive behavioral therapy, cognitive rehabilitation [65], psychotherapeutic interventions, social work interventions, self-management strategies [80], and specialized interdisciplinary rehabilitation [81] for reducing chronic post-concussive symptoms. In addition, future studies should explore whether a patient’s outcome after mTBI can be improved by removing or reducing a prognostic factor. For example, while we know that patients experiencing intense headaches or pain after mTBI are more likely to have poor long-term outcomes, more research is needed to examine the effects of intervention reducing this prognostic factor on persistent post-concussion symptoms outcomes.

It is possible that clinical decision-making and organizational interventions can be developed by taking into consideration these prognostic factors. For example, adopting more rigorous criteria to identify patients who would benefit from further treatment, such as identifying those who are at high risk of developing persistent symptoms by having ≥ 3 symptoms according to the RPQ during the early phase post-injury [74]. Rytter et al. [81] suggested a focus on the effective identification of patients who are at risk of maintaining persistent post-concussive symptoms in order to initiate a treatment plan in a timely fashion. Early and effective identification of risk factors of persistent symptoms may indicate earlier intervention and prevention of such chronicity after mTBI [64].

Evidence have highlighted the importance of early identification of this at-risk population and how to prevent persistent symptoms before onset. However, we need to acknowledge that some individuals may fall through the cracks of the system and they can experience a prolonged recovery (more than 3 months) [6, 7]. Once these patients show signs of chronic symptomatology, prognostic factors are therefore more than important to consider, as time is running out. The authors are unanimous that patients experiencing persistent consequences following mTBI must receive healthcare services in a timely manner [12, 81]. Referral to a specialized multidisciplinary mTBI clinic can be appropriate for patients with persistent symptoms that do not respond to treatment in a primary care setting [82]. Access to such specialized rehabilitation services can be complex and patients often face long waiting times [83–85]. The results of this OvR could help multiple stakeholders, such as clinicians and healthcare managers, to understand the prognosis of their patients and to focus their time and resources on patients needing them the most. It could also inform decision-makers and policymakers about the challenge of early identification of prognostic factors in order to prevent the onset of persistent symptoms.

It is known that overviews of reviews often lack methodological rigor because there are no pre-established reporting guidelines [86]. However, we have based our methods on Cochrane Handbook [29] and several previous works [30–35, 87] that can appropriately guide us through a rigorous process. We must acknowledge that our review has some limitations. The first is that, by the nature of an overview, we limited our data analysis to what was presented in the systematic reviews included. Thus, results presented in our OvSR had already been synthesized by previous authors, so prognostic factors may have been omitted as we did not analyze the primary articles. Our intent was to have a broad picture of all prognostic factors reviewed in the literature, and we are aware that our results should be interpreted with caution. Even if we showed a very small CCA value, we did not conduct further analysis regarding the primary studies in each review, so two reviews may have analyzed data from the same studies. While most of the included systematic reviews targeted only the adult population with mTBI, some have examined a broader population, which made it more difficult to extract the relevant data in some systematic reviews. We chose to include all adult populations with mTBI (veterans, military personnel, and adults with sport-related injury) to be in adequation with our objective, but it may have led to complicate the data synthesis of prognostic factors into a single comprehensive model. We also need to mention that few systematic reviews presented results of prognostic factors that did not demonstrate an association with outcomes, which shows several publication biases.

## Conclusion

Having multiple articles reviewing information about populations with mTBI and prognostic factors associated with persistent outcomes, we aimed to produce a synthesis of this extensive evidence. We found a broad portrait of prognostic factors related to persistent post-concussion symptoms, and this overview highlighted the need for a comprehensive picture of this condition. Premorbid characteristics as well as negative perceptions of the recovery need to have special attention regarding the risk of persistent symptoms. We believe that these prognostic factors would guide clinical decisions and research related to prevention and intervention regarding persistent post-concussion symptoms.

## Supplementary Information

**Additional file 1.**

**Additional file 2.**

**Additional file 3.**

**Additional file 4.**

## Data Availability

All data generated or analyzed during this study are included in this overview article.

## Abbreviations

ASD: Acute stress disorder
BAI/BDI: Beck Anxiety/Depression Inventory
CCA: Corrected covered area
CINAHL: Cumulative Index to Nursing and Allied Health Literature
CT: Computed tomography
DTI: Diffusion tensor imaging
DSM: Diagnostic and Statistical Manuel of Mental Disorders
ED: Emergency department
GCS: Glasgow Coma Scale
HR: High risk
HADS: Hospital Anxiety and Depression Scale
LOC: Loss of consciousness
LR: Low risk
MRI: Magnetic resonance imaging
MRS: Magnetic resonance spectroscopy
mTBI: Mild traumatic brain injury
OvSR: Overview of systematic reviews
PRISMA: Preferred Reporting Items for Systematic Reviews and Meta-Analyses
PCS: Postconcussion symptoms
PET: Positron emission tomography
PTA: Post-traumatic amnesia
PTSD: Post-traumatic stress disorder
ROBIS: Risk of Bias in Systematic Review
rs-fMRI: Resting-state functional magnetic resonance imaging
RTW: Return to work
WHO: World Health Organization
