# Supplementary material for: Prognostic factors for persistent symptoms in adults with mild traumatic brain injury: an overview of systematic reviews
Source: Syst Rev. 2023 Jul 20;12:127. doi: 10.1186/s13643-023-02284-4 (PMC10357711; doi:10.1186/s13643-023-02284-4)
Supplement: Supplementary file 2 — Additional file 2. [file 13643_2023_2284_MOESM2_ESM.docx]

Example of search strategy in Medline database

| 1) Concept : **MTBI concussion** | | | | |
| --- | --- | --- | --- | --- |
| #1 | [ti.] | | | (concuss* OR commotio* cerebr* OR cerebral* commotio* OR mtbi) or ((mild OR minor OR minimal) adj3 (traumatic brain OR tbi)) |
| #2 | [ab.] | | | (concuss* OR commotio* cerebr* OR cerebral* commotio* OR mtbi) OR ((mild OR minor OR minimal) adj3 (traumatic brain OR tbi)) |
| #3 | [Mesh] | | | Brain injuries, traumatic/ |
| #4 | [Mesh] | | | Brain concussion/ |
| #5 | #1 OR #2 OR #3 OR #4 | | | |
| 2) Concept : **Chronic/persistent symptoms** | | | | |
| #6 | | [ti.] | | (prolong* OR persist* OR unresolved OR delay* OR chronic* OR post-concuss* OR postconcuss*) |
| #7 | | [ab.] | | (prolong* OR persist* OR unresolved OR delay* OR chronic* OR post-concuss* OR postconcuss*) |
| #8 | | [Mesh] | | Post-Concussion Syndrome/ |
| #9 | | #6 OR #7 OR #8 | | |
| 3) Concept: **Prognosis** | | | | |
| #10 | | | [ti.] | (prognos* OR predict* OR course* OR outcome*) |
| #11 | | | [ab.] | (prognos* OR predict* OR course* OR outcome*) |
| #12 | | | [Mesh] | Prognosis/ |
| #13 | | | #10 OR #11 OR #12 | |
| #14 | | | #5 AND #9 AND #13 | |
| 4) Concept: **Systematic reviews** | | | | |
| #15 | | | [pt.] | Systematic review |
| #16 | | | **#14 AND #15** | |
