# Supplementary material for: Prognostic factors for persistent symptoms in adults with mild traumatic brain injury: an overview of systematic reviews
Source: Syst Rev. 2023 Jul 20;12:127. doi: 10.1186/s13643-023-02284-4 (PMC10357711; doi:10.1186/s13643-023-02284-4)
Supplement: Supplementary file 4 — Additional file 4. [file 13643_2023_2284_MOESM4_ESM.docx]

| Review | Concerns regarding specification of study eligibility criteria | Concerns regarding methods used to identify and/or select studies | Concerns regarding methods used to collect data and appraise studies | Concerns regarding methods used to synthesize results | Risk of bias in the review |
| --- | --- | --- | --- | --- | --- |
| Begaz (1) | Low | High | High | High | High |
| Belanger (2) | Low | High | High | Low | High |
| Belanger (3) | High | High | High | High | High |
| Biagianti (4) | Low | Low | High | Unclear | High |
| Boyle (5) | Low | Low | Low | Low | Low |
| Cancelliere (6) | Low | Low | Low | Low | Low |
| Cancelliere (7) | Low | Unclear | Unclear | High | High |
| Carroll (8) | Low | Low | Low | Low | Low |
| Carroll (9) | Low | Low | Low | Low | Low |
| Cassidy (10) | Low | Low | Low | Low | Low |
| Finkbeiner (11) | High | High | High | High | High |
| Godbolt (12) | Low | Low | Low | Low | Low |
| Khong (13) | High | Low | Low | High | High |
| King (14) | Unclear | Unclear | High | High | High |
| King (15) | Unclear | Unclear | High | High | High |
| Ludwig (16) | High | High | Low | Low | Low |
| Manley (17) | Low | High | Low | High | Low |
| Mercier (18) | Low | Low | Low | Low | Low |
| Mercier (19) | Low | Low | Low | Low | Low |
| Merritt (20) | High | High | High | High | High |
| Ofoghi (21) | High | Low | High | Low | High |
| Puig (22) | High | Low | Low | High | Low |
| Silverberg (23) | Low | High | High | Low | Low |
| Sullivan (24) | High | Low | High | High | High |
| Zhu (25) | High | High | High | High | High |

1. Begaz T, Kyriacou DN, Segal J, Bazarian JJ. Serum biochemical markers for post-concussion syndrome in patients with mild traumatic brain injury. Journal of Neurotrauma. 2006;23(8):1201-10.

2. Belanger HG, Curtiss G, Demery JA, Lebowitz BK, Vanderploeg RD. Factors moderating neuropsychological outcomes following mild traumatic brain injury: A meta-analysis. Journal of the International Neuropsychological Society. 2005;11(3):215-27.

3. Belanger HG, Spiegel E, Vanderploeg RD. Neuropsychological performance following a history of multiple self-reported concussions: a meta-analysis. Journal of the International Neuropsychological Society : JINS. 2010;16(2):262-7.

4. Biagianti B, Stocchetti N, Brambilla P, Van Vleet T. Brain dysfunction underlying prolonged post-concussive syndrome: A systematic review. Journal of Affective Disorders. 2020;262:71-6.

5. Boyle E, Cancelliere C, Hartvigsen J, Carroll LJ, Holm LW, Cassidy JD. Systematic review of prognosis after mild traumatic brain injury in the military: Results of the international collaboration on mild traumatic brain injury prognosis. Archives of Physical Medicine and Rehabilitation. 2014;95(3 SUPPL):S230-S7.

6. Cancelliere C, Kristman VL, Cassidy JD, Hincapié CA, Côté P, Boyle E, et al. Systematic review of return to work after mild traumatic brain injury: Results of the international collaboration on mild traumatic brain injury prognosis. Archives of Physical Medicine and Rehabilitation. 2014;95(3 SUPPL):S201-S9.

7. Cancelliere C, Donovan J, David Cassidy J. Is sex an indicator of prognosis after mild traumatic brain injury: A systematic analysis of the findings of the world health organization collaborating centre task force on mild traumatic brain injury and the international collaboration on mild traumatic brain injury prognosis. Archives of Physical Medicine and Rehabilitation. 2016;97(2):S5-S18.

8. Carroll LJ, Cassidy JD, Peloso PM, Borg J, von Holst H, Holm L, et al. Prognosis for mild traumatic brain injury: results of the WHO Collaborating Centre Task Force on Mild Traumatic Brain Injury. Journal of Rehabilitation Medicine Supplement. 2004;[36](43):84-105.

9. Carroll LJ, Cassidy JD, Cancelliere C, Côté P, Hincapié CA, Kristman VL, et al. Systematic review of the prognosis after mild traumatic brain injury in adults: Cognitive, psychiatric, and mortality outcomes: Results of the international collaboration on mild traumatic brain injury prognosis. Archives of Physical Medicine and Rehabilitation. 2014;95(3 SUPPL):S152-S73.

10. Cassidy JD, Cancelliere C, Carroll LJ, Côté P, Hincapié CA, Holm LW, et al. Systematic review of self-reported prognosis in adults after mild traumatic brain injury: Results of the international collaboration on mild traumatic brain injury prognosis. Archives of Physical Medicine and Rehabilitation. 2014;95(3 SUPPL):S132-S51.

11. Finkbeiner NWB, Max JE, Longman S, Debert C. Knowing what we don't know: Long-term psychiatric outcomes following adult concussion in sports. Canadian Journal of Psychiatry. 2016;61(5):270-6.

12. Godbolt AK, Cancelliere C, Hincapie CA, Marras C, Boyle E, Kristman VL, et al. Systematic review of the risk of dementia and chronic cognitive impairment after mild traumatic brain injury: results of the International Collaboration on Mild Traumatic Brain Injury Prognosis. Archives of Physical Medicine & Rehabilitation. 2014;95(3 Suppl):S245-56.

13. Khong E, Odenwald N, Hashim E, Cusimano MD. Diffusion tensor imaging findings in post-concussion syndrome patients after mild traumatic brain injury: A systematic review. Frontiers in Neurology. 2016;7(SEP).

14. King NS. A systematic review of age and gender factors in prolonged post-concussion symptoms after mild head injury. Brain Injury. 2014;28(13-14):1639-45.

15. King N. Permanent post concussion symptoms after mild head injury: A systematic review of age and gender factors. NeuroRehabilitation. 2014;34(4):741-8.

16. Ludwig R, D'Silva L, Vaduvathiriyan P, Rippee MA, Siengsukon C. Sleep Disturbances in the Acute Stage of Concussion are Associated With Poorer Long-Term Recovery: A Systematic Review. PM and R. 2020;12(5):500-11.

17. Manley G, Gardner AJ, Schneider KJ, Guskiewicz KM, Bailes J, Cantu RC, et al. A systematic review of potential long-term effects of sport-related concussion. British Journal of Sports Medicine. 2017;51(12):969-77.

18. Mercier E, Tardif PA, Cameron PA, Batomen Kuimi BL, Émond M, Moore L, et al. Prognostic Value of S-100β Protein for Prediction of Post-Concussion Symptoms after a Mild Traumatic Brain Injury: Systematic Review and Meta-Analysis. Journal of Neurotrauma. 2018;35(4):609-22.

19. Mercier E, Tardif P-A, Cameron PA, Émond M, Moore L, Mitra B, et al. Prognostic value of neuron-specific enolase (NSE) for prediction of post-concussion symptoms following a mild traumatic brain injury: a systematic review. Brain Injury. 2018;32(1):29-40.

20. Merritt VC, Padgett CR, Jak AJ. A systematic review of sex differences in concussion outcome: What do we know? The Clinical Neuropsychologist. 2019;33(6):1016-43.

21. Ofoghi Z, Dewey D, Barlow KM. A Systematic Review of Structural and Functional Imaging Correlates of Headache or Pain after Mild Traumatic Brain Injury. Journal of Neurotrauma. 2020;37(7):907-23.

22. Puig J, Ellis MJ, Kornelsen J, Figley TD, Figley CR, Daunis-I-Estadella P, et al. Magnetic Resonance Imaging Biomarkers of Brain Connectivity in Predicting Outcome after Mild Traumatic Brain Injury: A Systematic Review. Journal of Neurotrauma. 2020;37(16):1761-76.

23. Silverberg ND, Gardner AJ, Brubacher JR, Panenka WJ, Li JJ, Iverson GL. Systematic review of multivariable prognostic models for mild traumatic brain injury. Journal of Neurotrauma. 2015;32(8):517-26.

24. Sullivan KA, Kempe CB, Edmed SL, Bonanno GA. Resilience and Other Possible Outcomes After Mild Traumatic Brain Injury: a Systematic Review. Neuropsychology Review. 2016;26(2):173-85.

25. Zhu J, Ling J, Ding N. Association between Diffusion Tensor Imaging Findings and Cognitive Outcomes Following Mild Traumatic Brain Injury: A PRISMA-Compliant Meta-Analysis. ACS chemical neuroscience. 2019;10(12):4864-9.
